# Relationship between Human Pupillary Light Reflex and Circadian System Status

**DOI:** 10.1371/journal.pone.0162476

**Published:** 2016-09-16

**Authors:** Maria Angeles Bonmati-Carrion, Konstanze Hild, Cheryl Isherwood, Stephen J. Sweeney, Victoria L. Revell, Debra J. Skene, Maria Angeles Rol, Juan Antonio Madrid

**Affiliations:** 1 Chronobiology Laboratory, Department of Physiology, Faculty of Biology, University of Murcia, IMIB-Arrixaca, 30100, Espinardo, Murcia, Spain; 2 Advanced Technology Institute and Department of Physics, University of Surrey, Guildford, Surrey, GU2 7XH, United Kingdom; 3 Chronobiology, Faculty of Health and Medical Sciences, University of Surrey, Guildford, Surrey, GU2 7XH, United Kingdom; Dalhousie University, CANADA

## Abstract

Intrinsically photosensitive retinal ganglion cells (ipRGCs), whose photopigment melanopsin has a peak of sensitivity in the short wavelength range of the spectrum, constitute a common light input pathway to the olivary pretectal nucleus (OPN), the pupillary light reflex (PLR) regulatory centre, and to the suprachiasmatic nuclei (SCN), the major pacemaker of the circadian system. Thus, evaluating PLR under short wavelength light (λ_max_ ≤ 500 nm) and creating an integrated PLR parameter, as a possible tool to indirectly assess the status of the circadian system, becomes of interest. Nine monochromatic, photon-matched light stimuli (300 s), in 10 nm increments from λ_max_ 420 to 500 nm were administered to 15 healthy young participants (8 females), analyzing: i) the PLR; ii) wrist temperature (WT) and motor activity rhythms (WA), iii) light exposure (L) pattern and iv) diurnal preference (Horne-Östberg), sleep quality (Pittsburgh) and daytime sleepiness (Epworth). Linear correlations between the different PLR parameters and circadian status index obtained from WT, WA and L recordings and scores from questionnaires were calculated. In summary, we found markers of robust circadian rhythms, namely high stability, reduced fragmentation, high amplitude, phase advance and low internal desynchronization, were correlated with a reduced PLR to 460–490 nm wavelengths. Integrated circadian (CSI) and PLR (cp-PLR) parameters are proposed, that also showed an inverse correlation. These results demonstrate, for the first time, the existence of a close relationship between the circadian system robustness and the pupillary reflex response, two non-visual functions primarily under melanopsin-ipRGC input.

## Introduction

In addition to rod and cone photoreceptors, the retina contains a small subset of retinal ganglion cells that express the photopigment melanopsin and are intrinsically photosensitive (ipRGCs) [1–9]. The axons of these ipRGCs project to several regions in the brain, such as the suprachiasmatic nuclei (SCN, the master circadian pacemaker), primarily through the retino-hypothalamic tract (RHT); the intergeniculate leaflet (IGL, a centre for circadian entrainment); the olivary pretectal nucleus (OPN, a control centre for the pupillary light reflex, PLR); the ventral subparaventricular zone (vSPZ, implicated in “negative masking” or acute suppression of locomotor activity by light in nocturnal animals); and the ventrolateral preoptic nucleus (VLPO, a control centre for sleep), among others [3,6,7,10,11]. Since the light input pathway for both the SCN and the OPN (PLR) relies on the ipRGCs, the PLR constitutes, under certain light conditions, a possible tool to examine, not only the effects of different light sources on the SCN pacemaker, but also the integrity and functional status of this input pathway to the central SCN clock and its relationship with the circadian system status. The situation, however, is more complicated, since ipRGCs also receive rod and cone inputs [12,13], integrating the extrinsic light input pathway.

Human retinal photoreceptors possess different wavelength sensitivities: λ_max_ 498 nm for rods, λ_max_ 440 nm for S-cones, λ_max_ 540 nm for M-cones, λ_max_ 580 nm for L-cones [14], and λ_max_ 480 nm for ipRGCs (melanopsin containing ganglion cells) [15]. Intensity thresholds for each photoreceptor in primates also differ, being higher for ipRGCs (~10–11 log quanta/cm^2^/s) [16,17] than for the remaining photoreceptors (cones 2.30 log quanta /cm^2^/s; rods 1.70 log quanta /cm^2^/s, at the cornea level [18]).

The dynamics of the PLR follows a general pattern, that can be influenced by the intensity, duration and spectral composition of the light. When a light stimulus is turned on, a high-velocity pupil constriction ensues until it reaches a minimum pupil size (maximal constriction amplitude). This early transient response is followed by pupillary redilation (escape) to a more sustained state of partial pupil constriction, which continues until the end of the light stimulus [19]. Studies in primates and humans suggest that the early transient pupil constriction under photopic conditions is predominantly a cone-driven response, while the sustained and persistent (post-illumination pupil response, PIPR) pupil constriction seem to be controlled by the melanopsin mediated intrinsic response [15,20,21], although recent studies have revealed that the outer retinal photoreceptors also contribute to sustained firing during long duration light stimuli [16,22,23]. Thus, analysis of the transient, sustained and persistent (or PIPR) pupillary response to light stimuli of different wavelengths, intensities and durations may be a useful tool to independently assess rod and cone function and the intrinsic activation of ipRGCs [19].

The possibility of assessing each photoreceptor contribution will, eventually, lead to identifying the precise retinal-SCN pathway activated by different light stimuli. The extrinsic light signal is also relevant in circadian photoentrainment and affects circadian functioning, although the exact photoreceptor contribution has not yet been established. Inadequate light exposure, either in intensity, timing or spectral composition, generates chronodisruption or internal desynchrony (for a review, see [24]). However, assessing misalignment among rhythms requires simultaneous measurements of several circadian outputs, as well as circadian inputs. In this sense, ambulatory circadian monitoring (ACM), based on wrist actigraphy, thermometry and body position measurements that allows continuous recordings over longer periods of time (up to several weeks) has proven useful, not only for evaluating rhythm stability across different days [25–27], but also to establish circadian phase in humans under free living conditions [26]. Due to the common pathway between the PLR and circadian entrainment, it seems reasonable to hypothesize that a relationship between the global pupillary response and circadian system status could exist. If this were the case, pupillometry could constitute a suitable technique to predict the circadian system status, with clinical applications such as assessing potential efficacy of light therapy. However, this possibility, with the exception of seasonal affective disorder (SAD) (for a review, see [28]), has not been explored.

Therefore, the aim of this study was to evaluate the possible interrelationship between the pupillary response, considered globally through an integrated PLR parameter, and the circadian input and output signals such as wrist temperature rhythm, motor activity and light exposure patterns monitored under free living conditions, also integrating these aspects into a global parameter. Due to the SCN/OPN common pathway, we hypothesized a direct relationship between the global pupillary response and an integrated circadian system parameter, with greater pupil responses corresponding with a more robust circadian status.

## Materials and Methods

### Participants

Participants were 15 healthy non-smoking volunteers (8 women) between 19 and 35 years of age (25.7 ± 2.3 years, mean ± SEM). All of them had no medical or mental disorders and were not taking any medication that could affect circadian rhythms, as determined from general health questionnaires completed during the screening period. None of the participants were shift workers nor had crossed more than two time zones in the two months prior to study admission. They had regular sleep-wake cycles with no reported sleep disorders (Pittsburgh Sleep Quality Index ≤ 5) [29], and were neither extreme morning nor evening types (Horne-Östberg Morningness-Eveningness Questionnaire score: 55.3 ± 3.1; mean ± SEM) [30]. A full ophthalmic examination including uncorrected vision, near vision corrected, ophthalmoscopy, pupil reactions, Henson Field Test, Refraction, Intra-Ocular Pressure, Oculomotor Status, Stereo Acuity, Accommodation and Color vision by the Ishihara test, was performed to confirm they all were free from any ocular disorders.

Volunteers received appropriate information about the study protocol, signed a written informed consent form before being enrolled into the study and were compensated for their participation. This research project was approved by the University of Surrey Ethics Committee and abides by the principles set out by the Declaration of Helsinki.

### Ambulatory circadian monitoring (ACM)

The ACM protocol used was similar to that previously described [27] with participants keeping their normal life style for 9 days, beginning two weeks prior to the pupillometry sessions. They wore an actimetry device that provides actigraphic and light exposure data (AWL, Cambridge Neurotechnology, UK), and a temperature data logger (Thermochron iButton DS1921H, Dallas, Maxim, Dallas, TX) to assess the wrist temperature (WT) rhythm. This sensor has a sensitivity of 0.125°C and was programmed to sample once every 10 minutes. It was attached to a double-sided cotton sport wristband, and the sensor surface was positioned on the radial artery of the non-dominant hand, as previously described [31].

Besides, the previous week to pupillometry sessions, participants were required to maintain a regular sleep/wake schedule. For 72 h before each laboratory session and during it, participants refrained from caffeinated drinks, alcohol, excessive exercise, bright lights and non-steroidal anti-inflammatory drug intake.

### Questionnaires

For assessment of diurnal preference, participants were required to complete the Horne-Östberg (HO) Morningness-Eveningness Questionnaire [30]. In addition, participants also completed the Pittsburgh Sleep Quality Index (PSQI) to determine their sleep quality [29] and the Epworth Sleepiness Scale (ESS) for assessment of daytime sleepiness [32].

### In-laboratory protocol

**Fig 1:**
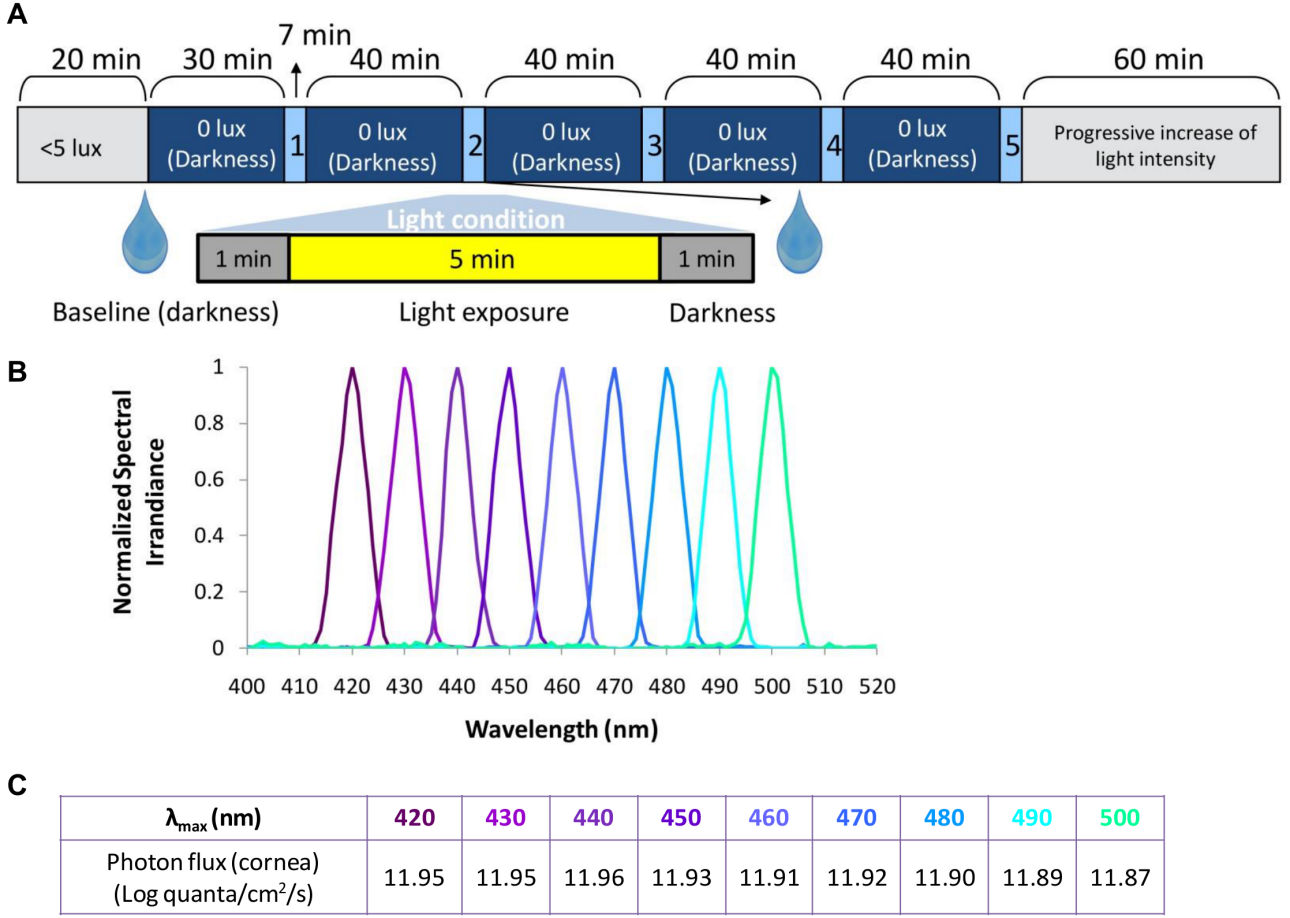
In-laboratory protocol. (A) Light conditions tested including the study protocol, (B) normalized spectra and (C) their corresponding photon fluxes at the cornea. Drop symbol shows the time when the pupil dilator, tropicamide, was applied.

**Fig 2:**
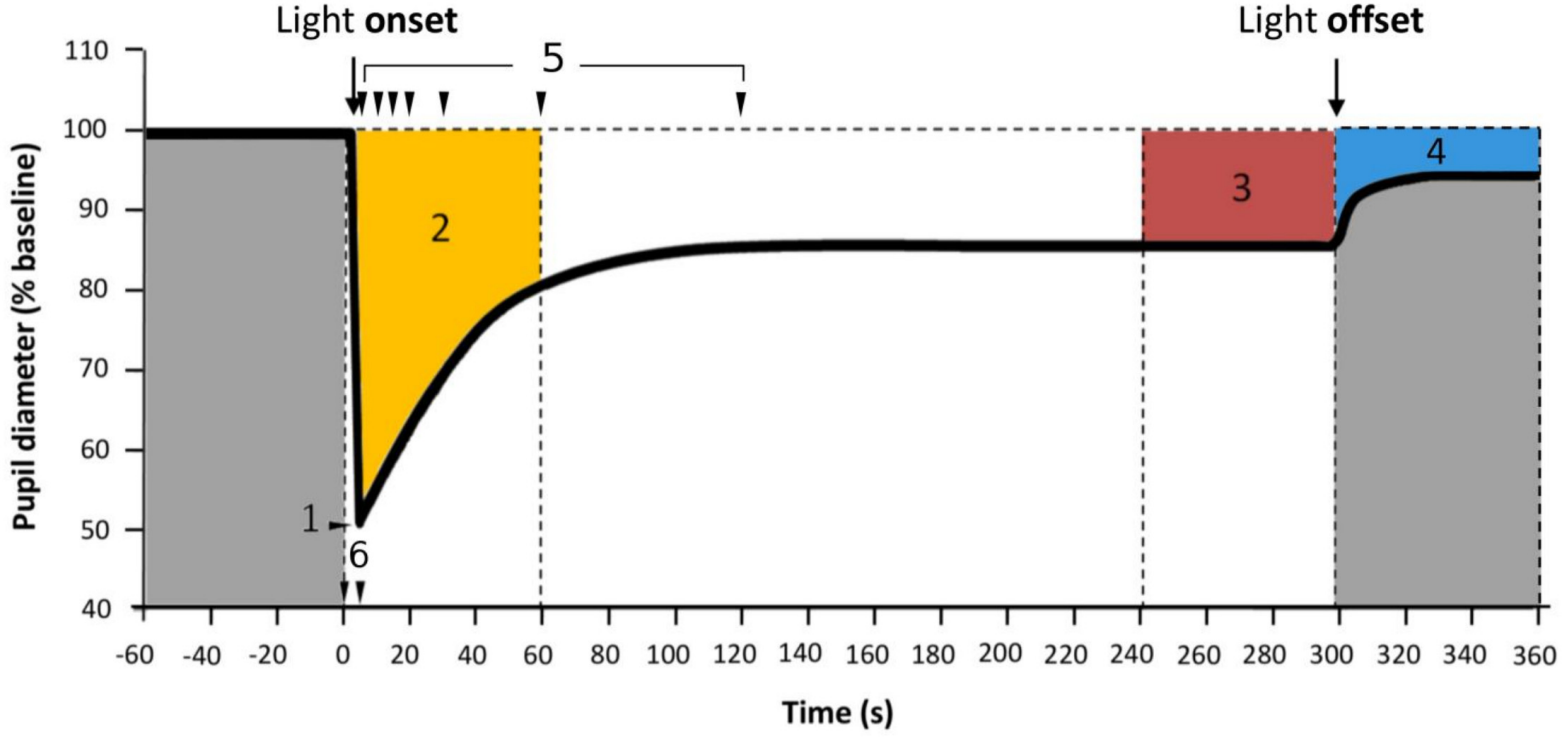
Parameters assessed for PLR. 1: Minimum diameter, expressed as percentage constriction; 2: Area Under the Curve from 0 to 60 seconds of light exposure (AUC_60_, orange); 3: Area Under the Curve from 240 to 300 seconds of light exposure (AUC_240_, in red); 4: Area Under the Curve from light offset to the end of the recording (from 300 to 360 seconds of recording) (AUC_300_, blue); 5: Percentage pupil constriction. Each arrow indicates, from left to right, TL5, TL10, TL15, TL20, TL30, TL60 and TL120, respectively, thus the percentage of pupil constriction at 5, 10, 15, 20, 30, 60 and 120 seconds after light onset.; 6: Time from light onset to the minimum pupil diameter reached during pupil constriction.

A randomised, within-subject design was conducted. Full details of the study protocol are presented in Fig 1A. The participants arrived at the laboratory and stayed seated in dim light (< 5 lux) for 20 minutes, prior to receiving a drop of the pupil dilator, tropicamide (Minims Tropicamide (1.0%), Chauvin Pharmaceuticals, Romford, UK), in the right eye. After that, the participant remained in darkness (0 lux + eye mask) for 30 minutes until the first light condition was tested. Each light condition consisted of a 5-minute light stimulus to the right eye for each wavelength studied (see Figs 1 and 2, and section “Light exposure for PLR assessment”). Following this, the participant again remained in total darkness for 40 minutes, prior to receiving the second light stimulus. Immediately after this light condition, a second drop of tropicamide (Minims Tropicamide (1.0%)) was administered into the right eye. These steps, except the tropicamide administration, were followed until five light conditions were completed.

The left pupil (undilated) was recorded for one minute in darkness in order to obtain a baseline measurement to normalize the pupil diameter under light exposure. Following this, the dilated pupil (right eye) received the 5-minute light pulse, during which the left pupil diameter was recorded. After light offset, the pupil was recorded for another minute in darkness. All participants came to the laboratory twice, receiving nine different light conditions in total (the 460 nm light stimulus being administered twice, once per laboratory session). The order of presentation of the different light conditions was randomized for each laboratory session. Sessions always started at the same time for each subject (10:00–10:30h).

### Light exposure for PLR assessment

The five minute light pulse was administered onto the participant's right eye using a specially constructed 45 cm diameter Ganzfeld sphere (Apollo Lighting, Leeds, UK). The sphere was coated with white reflectance paint (WRC-680 Labsphere, Pro-Lite Technology, Bedfordshire, UK) and was illuminated via a fiber optic cable connected to light box housing an ultra high-pressure mercury lamp (Focus 100LS3, 100 W, Philips Lighting, Eindhoven, The Netherlands) to provide patternless illumination [33]. A black barrier was placed at the front of the sphere in order to avoid any light input onto the left eye (which was recorded by the video camera).

Monochromatic lights (λ_max_ 420, 430, 440, 450, 460, 470, 480, 490 and 500 nm) each with a half maximal bandwidth λ_0.5_ of 7 nm were obtained using a Bentham M300 monochromator (Fig 1B). Light from the light box was focused onto the monochromator slits and the monochromator output focussed back onto the fiber optic cable to be delivered into the sphere. The achieved photon density was approximately 11.9 log quanta at the level of the cornea (Fig 1C), and approximately 11.6 log quanta considering optical media attenuation estimation (-0.3 log quanta). Due to technical limitations, the same photon fluxes could not be achieved for all the wavelengths tested. This range of wavelengths was chosen according to previous bibliographic findings on human circadian system short wavelength sensitivity [34–38]. Studying the PLR under wavelengths shorter than the melanopsin λ_max_ peak could also clarify the role of very short wavelength light.

### Pupil recording

A pupillometer system was used to record the consensual pupillary constriction response. The pupil size was recorded from the infrared illuminated (left) eye using an infrared video pupil tracking system (ViewPoint Eye Tracker^®^; Arrington Research Inc., Scottsdale, AZ). A video camera and an infrared illuminator were set up in a plane parallel to the cornea and to the left of the integrating sphere. The participants were seated in front of the sphere in darkness, placing their head on the support provided with the pupillometer system. The left eye was focused by the infrared camera and the participant was instructed to stare at a red spot located just behind the camera. The researcher helped the participants to comfortably achieve the correct position and they were asked to blink and move as little as possible. The system recorded 220 data per second.

### Data analysis

#### Pupillary light reflex (PLR)

- **Data processing**: pupil diameter was analysed using software specifically designed by the Chronobiology Laboratory (University of Murcia) for this purpose (PupiLabWare^®^). The PLR generates a large amount of data due to the high sample frequency (220 Hz) that must be processed. Since filtering data to eliminate artefacts and data processing are highly time consuming tasks, specific software, based on Java language was implemented. It easily imports data from the video tracking system and allows blink artefacts to be eliminated by filtering the raw data. Afterwards, data can be re-sampled at the necessary frequency (100 Hz to obtain some parameters and 10 Hz to average all the recordings obtained under the same light condition and to reduce noise). The pupil diameter was then individually normalized according to the 60 second baseline measurement obtained from each participant (Fig 1A), expressed as a percentage of the baseline.
- **Determination of baseline and normalized pupil size:** For each test session, a baseline pupil size, was calculated as the mean diameter during 60 seconds in darkness before light stimulation. The pupil size was calculated as a normalized pupil size (NPS), i.e., ratio of the measured pupil diameter divided by the baseline pupil size.
- **Main pupil outcome parameters**: all the parameters were obtained from the normalized waveforms, with respect to the baseline pupil diameter (Fig 2).
  - Minimum diameter expressed as relative maximum rapid pupil constriction under each light stimulus: the relative maximum pupil constriction achieved after the light onset, calculated as $\left[\frac{\left(\text{baseline }-\text{diameter}\right)}{\text{baseline}}\right]\times 100$.
  - Time to minimum ("response time") (Fig 2): time required to achieve the minimum pupil diameter or relative maximum pupil constriction.
  - Velocity of pupil constriction: calculated as $\frac{100-\text{minimum diameter},\text{ without inversion})}{\text{time to minimum}}$.
  - Area under the curve (AUC) (Fig 2). The AUC was calculated as follows: $\text{AUC}=(\text{AUC}\Sigma_{t0}^{t1}100-\text{NPS}$), where t_0_ is the initial time point of pupil response and t_1_ is the end time, 100 is the baseline pupil size, and NPS is the normalized pupil size. Three AUCs were calculated: AUC_60_, that corresponds to the first minute of light exposure; AUC_240_, that corresponds to the last minute of light exposure; and AUC_300_, that corresponds to the minute after light offset.
  - TL(5, 10, 15, 20, 30, 60, 120) (Fig 2): Pupil diameter expressed as pupil constriction at those fixed time points (in seconds) of the PLR recording (after light onset). These were calculated as the mean diameter of the immediately previous and subsequent second (thus, each measure originally consisted of 440 frames).

In order to obtain a global parameter for the PLR, we normalized the values for the minimum pupil diameter (Min) and the AUC_240_ (i.e. one parameter reflecting the transient and the other reflecting the sustained part of the PLR), and averaged them *per* participant and wavelength. These normalized values were averaged from 460 to 490 nm to obtain the “circadian photoreception-PLR” parameter (cp-PLR).

### Wrist temperature, wrist acceleration and light exposure rhythm analysis

Firstly wrist temperature (WT) data were filtered in order to eliminate artefacts such as those produced by temporarily removing the sensors (for example for personal hygiene). For the chronobiological analysis, wrist temperature (WT), wrist acceleration (WA) and the light exposure pattern (L) data were characterized using non-parametric, cosinor and Fourier analysis. Non-parametric analysis [39] was used to calculate the following phase markers: the mid-point time of the ten hours with the lowest (L10) values for WT, or highest values (M10) for WA and L, and the mid-point time of the five consecutive hours with the highest (M5) values for WT, and lowest values (L5) for WA and L. Interdaily stability (IS), intradaily variability (IV), relative amplitude (RA) and the circadian function index (CFI) were also calculated to characterize the circadian pattern for each variable, as previously described [25,39,40]. The mean values at L10/M10 and M5/L5 were also calculated (VL10/VM10 and VM5/VL5). Rhythm parameters estimated from the cosinor procedure included mesor (24 h rhythm-adjusted mean of the cosine curve fitted to the data), amplitude (difference between the maximum and the cosine calculated mesor) and acrophase (timing of the peak of the fitted cosine curve).

From the Fourier analysis, the first (Pot_1_), and the accumulative power of the first twelve harmonics (Pot_1-12_) were obtained and its ratio (Pot_1/1-12_) calculated in order to obtain the circadianity index [41].

Internal desynchronization indexes were also calculated between WT and WA (WT/WA), WT and light exposure pattern (WT/L) and WA and L (WA/L) by means of the following formulae:

$$DI\left(WT/WA\right)=\frac{\left|M5_{WT}-L5_{WA}\right|}{12}$$

$$DI\left(WT/L\right)=\frac{\left|M5_{WT}-L5_{L}\right|}{12}$$

$$DI\left(WA/L\right)=\frac{\left|L5_{WA}-L5_{L}\right|}{12}$$

In order to obtain an integrated parameter containing information about the robustness, timing and the variable level of the participants’ rhythms, we first normalized the values for M5 (for WT), L5 (for WA and L), VM5 (for WT), VL5 (for WA and L) and the ratio (Pot_1/1-12_) for the three variables, and the Horne-Östberg and Pittsburgh Sleep Quality Index scores. Then, M5, L5, VL5 and PSQI score were inverted since higher values indicate worse circadian system status. All these parameters were then averaged for each participant, obtaining the circadian status index (CSI).

### Statistical analysis

All statistical analyses were carried out using SPSS 15.0 (SPSS Inc., Chicago, IL, USA). For both PLR and ACM analysis, the test used was one- or two-way repeated measures ANOVA. Paired T-test was used when comparing AUC_60_ and AUC_240_. In order to evaluate the relationship between the PLR and circadian parameters, linear correlations were performed between both of them. Correlations were considered statistically significant when *p* < 0.05. Bonferroni correction for multiple comparisons was performed. Correlations calculated between global parameters (CSI and cp-PLR, previously defined) imply a reduction in comparisons performed.

## Results

**Fig 3:**
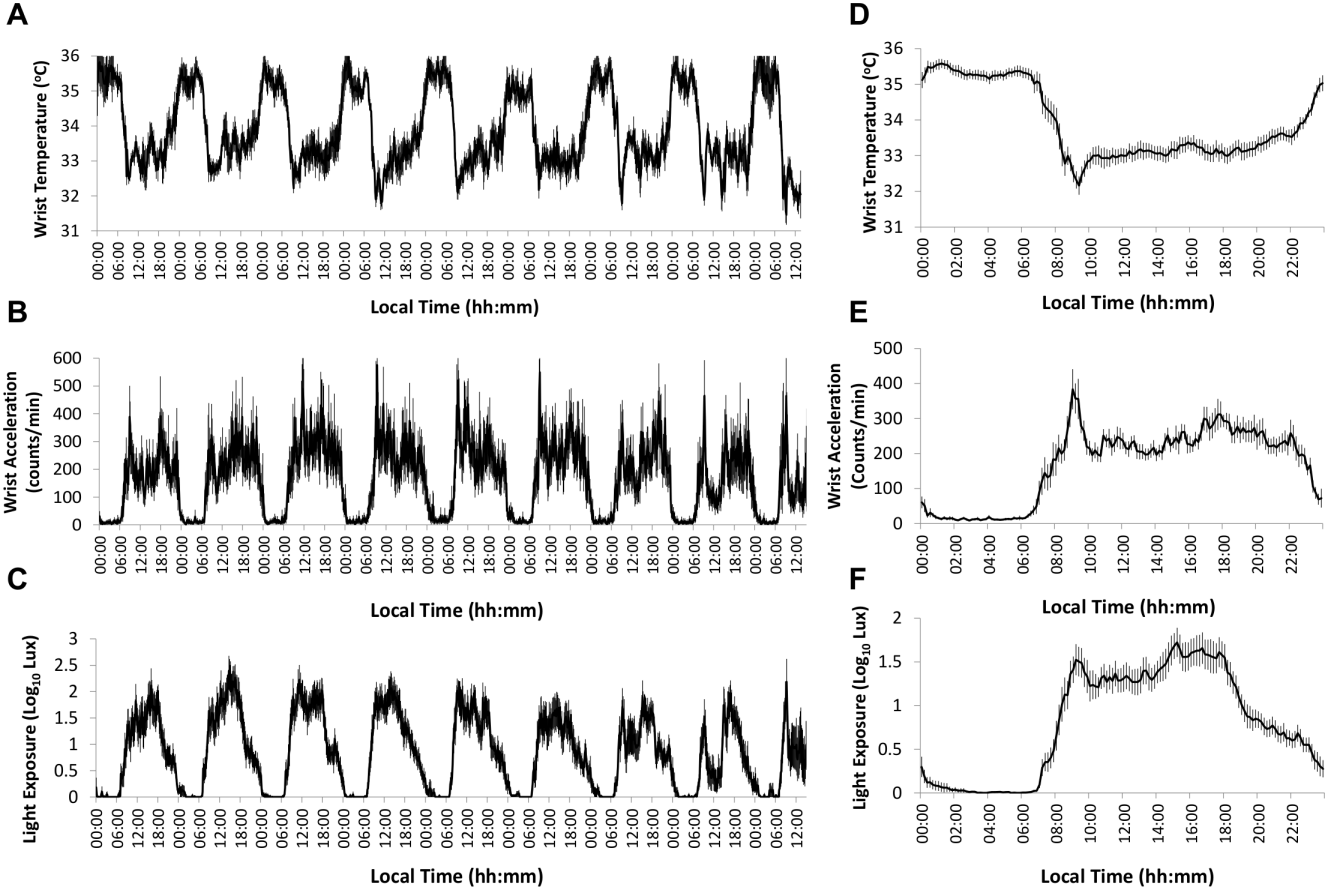
Ambulatory circadian monitoring. Left panel: nine-day averaged recording for (A) wrist temperature, (B) wrist acceleration, and (C) light exposure, from 15 subjects. Right panel: averaged mean waveforms (n = 15) for (D) wrist temperature, (E) wrist acceleration, and (F) light exposure. Data are expressed as mean ± SEM.

The 9-day average wrist temperature (A), wrist acceleration (B) and light exposure pattern (C) for the study population, and their corresponding weekly mean waveforms are represented in Fig 3. As expected, higher values for wrist temperature were found at night, declining during the day. The opposite was found for wrist acceleration and light exposure, with higher values during the daytime. Cosinor, non-parametric and Fourier analysis were also performed on the wrist temperature, wrist acceleration and light exposure patterns (results shown in Table 1). The mean score (±SEM) for the Horne-Östberg (HO) Morningness-Eveningness Questionnaire was 55.3 ± 3.1, 3.5 ± 0.3 for the Pittsburgh Sleep Quality Index (PSQI), and 5.3 ± 0.9 for the Epworth Sleep Scale questionnaire. All these scores were within normal values for all participants included in the study.

**Table 1:** Cosinor, non-parametric and Fourier analysis for wrist temperature rhythm (WT), motor activity (wrist acceleration, WA) and light exposure (L) patterns (n = 15).

	Parameter	Wrist Temperature (WT)	Wrist Acceleration (WA)	Light exposure (L)
Cosinor	Mesor	33.83 ± 0.12	169.48 ± 10.34	0.79 ± 0.06
	Amplitude	1.38 ± 0.15	126.40 ± 9.95	0.81 ± 0.06
	Acrophase	3.97 ± 1.43	15.33 ± 0.27	14.64 ± 0.12
	Rayleigh	0.78 ± 0.06	0.90 ± 0.02	0.87 ± 0.03
	% of rhythm	33.87 ± 3.45	9.87 ± 1.01	27.41 ± 2.33
NPI	IS	0.54 ± 0.03	0.29 ± 0.01	0.40 ± 0.03
	IV	0.22 ± 0.03	0.45 ± 0.01	0.10 ± 0.01
	RA	0.04 ± 0.00	0.92 ± 0.01	0.99 ± 0.01
	CFI	0.60 ± 0.03	0.65 ± 0.01	0.77 ± 0.01
	L10/M10	13.71 ± 0.46	15.10 ± 0.64	13.88 ± 0.29
	M5/L5	3.29 ± 0.35	3.05 ± 0.20	2.82 ± 0.29
	VL10/VM10	32.95 ± 0.21	268.59 ± 15.71	1.44 ± 0.10
	VM5/VL5	35.41 ± 0.10	9.71 ± 1.16	0.01 ± 0.00
Fourier	Pot_1_	1.08 ± 0.20	8681.02 ± 1212.96	0.33 ± 0.05
	Pot _1–12_	1.54 ± 0.25	14910.24 ± 1879.87	0.40 ± 0.05
	Pot_1/1-12_	0.64 ± 0.04	0.58 ± 0.04	0.82 ± 0.01

NPI: non-parametric indexes (IS, interdaily stability; IV, intradaily variability; RA, relative amplitude; CFI, circadian function index; L10, the mid-point time of the ten consecutive hours with the lowest values; M10, the mid-point time of the ten consecutive hours with the highest values; M5, the mid-point time of the five consecutive hours with the highest values; L5, the mid-point time of the five consecutive hours with the lowest values; VL10 value for L10; VM10, value for M10; VM5, value for M5; VL5, value for L5).

**Fig 4:**
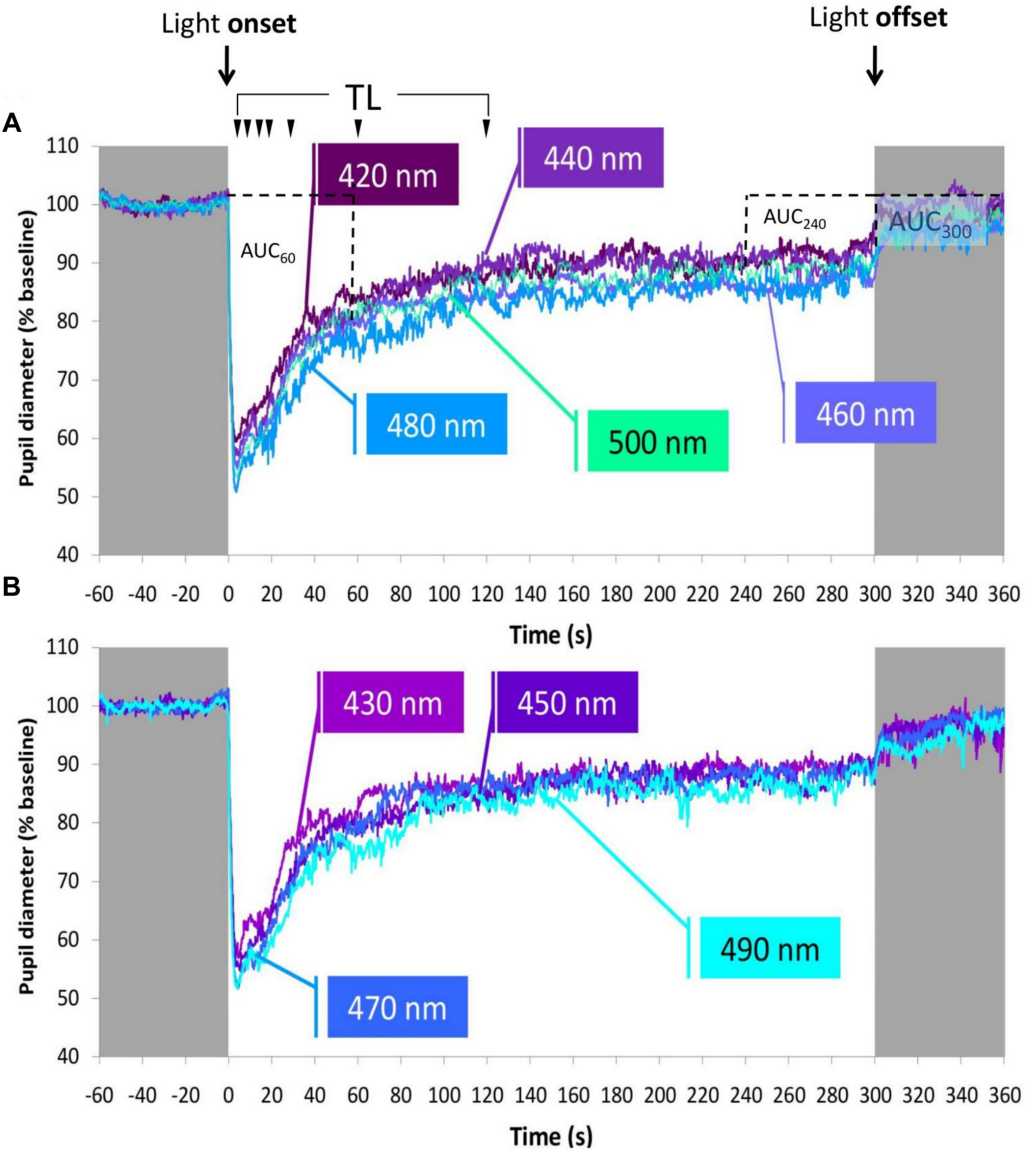
Averaged pupil recordings under the different lights tested (n = 15). Averaged pupil recordings under (A) 420, 440, 460, 480, 500 nm and (B) 430, 450, 470 and 490 nm lights. Grey areas represent darkness. On the top, light onset and offset are indicated. Metrics (see Fig 2) are also indicated here. TL: Each arrow indicates, from left to right, TL5, TL10, TL15, TL20, TL30, TL60 and TL120, respectively, and the percent baseline pupil diameter at 5, 10, 15, 20, 30, 60 and 120 seconds after light onset. AUC_60_ (Area Under the Curve from 0 to 60 seconds of light exposure), AUC_240_ (Area Under the Curve from 240 to 300 seconds of light exposure) and AUC_300_ (Area Under the Curve from 300 to 360 seconds of recording, after light offset in darkness) are indicated in Fig 4A.

Fig 4 shows the PLR under the different wavelengths averaged for all participants. The whole group PLR parameters are included in Tables 2 and 3. Note how wavelengths around 460–490 nm produced more marked transient and sustained responses, according to the minimum diameter (Min) or the Area Under the Curve from 0 to 60 (AUC_60_) and 240 to 300 (AUC_240_) seconds. However, the Area Under the Curve was different when comparing the first (AUC_60_) and last minute (AUC_240_) for all tested wavelengths (paired T-test, *p* < 0.05), demonstrating pupil diameter recovery throughout the light exposure.

**Table 2:** Averaged PLR parameters (n = 15) (I).

Wavelength (nm)	Min diameter (%constriction)	AUC_60_ (AU)	AUC_240_ (AU)	AUC_300_(AU)	Time to min (s)	Velocity (%/s)
**420**	46.0 ± 1.7	1517 ± 141	567 ± 100	153 ± 125.1	4.6 ± 0.5	10.4 ± 1.2
**430**	42.7 ± 1.9	1604 ± 151	647 ± 95	248 ± 61.0	4.2 ± 0.5	9.9 ± 1.1
**440**	46.8 ± 2.3	1696 ± 154	543 ± 127	68 ± 81.5	4.1 ± 0.4	12.2 ± 1.4
**450**	48.2 ± 1.8	1780 ± 150	700 ± 118	265 ± 73.3	4.6 ± 0.5	11.5 ± 1.2
**460**	48.4 ± 1.8	1841 ± 131	880 ± 131°	310 ± 110.2	4.3 ± 0.4	11.4 ± 1.0
**470**	50.2 ± 1.6*	1888 ± 129^#^^§^	704 ± 112	237 ± 77.5	4.4 ± 0.1	11.8 ± 0.7
**480**	49.9 ± 1.8*	1964 ± 146^#^	838 ± 100°	300 ± 61.6	3.7 ± 0.2	14.3 ± 0.9*
**490**	49.4 ± 1.7*	2007 ± 147^§^	794 ± 119	339 ± 100.8	3.8 ± 0.4	14.2 ± 1.2
**500**	49.7 ± 1.2*	1778 ± 108	656 ± 786010	193 ± 77.5	4.0 ± 0.3	13.5 ± 1.2

Min diameter, minimum pupil diameter achieved under each wavelength, expressed as % constriction; AUC_60_, Area Under the Curve from 0 to 60 seconds of light exposure; AUC_240_, Area Under the Curve from 240 to 300 seconds of light exposure; AUC_300_, Area Under the Curve from light offset to the end of the recording (from 300 to 360 seconds of recording); Time to min, Time from light onset to the minimum pupil diameter reached during constriction; Velocity, velocity of constriction.

*indicate statistically significant differences in Min diam between 430 nm and remaining wavelengths (Bonferroni *post hoc*, *p* < 0.05).

^#^ indicates statistically significant differences (Bonferroni *post hoc*, *p* < 0.042) in AUC_60_ *vs* 420 nm and

^§^ AUC_60_ *vs*. 430 nm.

**°** indicates a trend (Bonferroni *post hoc*, *p* < 0.056) in AUC_240_ *vs* 420 nm.

**Table 3:** Averaged PLR parameters (n = 15) (II).

Wavelength (nm)	TL5 (%constriction)	TL10 (%constriction)	TL15 (%constriction)	TL20 (%constriction)	TL30 (%constriction)	TL60 (%constriction)	TL120 (%constriction)
**420**	39.6 ± 2.3	35.5 ± 2.8	35.0 ± 2.8	31 ± 3.1	23.5 ± 2.8	16.4 ± 2.4	8.8 ± 1.6
**430**	39.7 ± 2.8	36.2 ± 3.0	37.9 ± 3.0	33.8 ± 3.7	20.9 ± 3.0	18.2 ± 3.0	12.9 ± 2.0
**440**	41.9 ± 2.3	37.5 ± 2.1	39.3 ± 2.0	34.3 ± 1.9	26.2 ± 3.1	18.9 ± 3.5	6.9 ± 2.1
**450**	44.4 ± 2.0	41.0 ± 2.2	41.2 ± 2.1	36.6 ± 2.9	29.1 ± 3.4	19.8 ± 2.2	13.1 ± 2.8
**460**	45.4 ± 2.2	41.7 ± 2.7	40.0 ± 2.4	36.3 ± 2.0	27.1 ± 3.0	18.7 ± 2.6	13.5 ± 1.9
**470**	47.8 ± 1.8	42.4 ± 2.2	43.5 ± 2.1	39.5 ± 2.5	29.7 ± 2.8	20.9 ± 2.9	12 ± 1.7
**480**	45.6 ± 1.9	44.2 ± 2.1	40.3 ± 2.5	39.2 ± 2.6	31.5 ± 3.9	22.8 ± 3.7	15.8 ± 2.3
**490**	45.0 ± 2.0	41.1 ± 2.2	41.3 ± 2.8	38.3 ± 2.6	29.8 ± 3.0	22 ± 3.1	14.4 ± 3.0
**500**	45.2 ± 1.7	40.8 ± 2.0	40.3 ± 1.7	35.5 ± 2.3	27.4 ± 2.7	17.7 ± 1.8	13.6 ± 2.4

TL5, TL10, TL15, TL20, TL30, TL60 and TL120, constriction at 5, 10, 15, 20, 30, 60 and 120 seconds after light onset, respectively.

In order to limit the number of correlations between the circadian and PLR parameters and avoid redundancies, from the circadian analysis only one parameter for each rhythm aspect was selected: level (VM5/VL5), robustness (Pot_1_/Pot_1-12_), and timing (M5/L5), and just three parameters from pupillometry: minimum diameter (Min), area under the curve during the first (AUC_60_) and last (AUC_240_) minute of light exposure (Table 4). As can be observed, parameters from the transient (AUC_60_) and sustained (AUC_240_) response were found to be correlated with the level, robustness and timing circadian parameters from the variables studied. For wrist temperature, only M5 was positively correlated with the minimum pupil diameter (Min) at 500 nm (thus, the later the time of M5, the greater the pupil constriction at the transient part of the PLR).

**Table 4:** Correlation analysis between PLR and different circadian parameters for wrist temperature rhythm (WT), wrist acceleration (WA) (circadian system output signal) and light exposure pattern (input signal).

		PLR correlation			
Variable	Circadian Parameter	PLR parameter	Wavelength (nm)	R	p
WT	M5	Min	500	0.547	0.043
		*AUC_240_	420	0.737	0.003
		AUC_240_	440	0.598	0.031
		AUC_60_	450	0.520	0.047
		AUC_240_	450	0.657	0.011
		Min	460	0.595	0.019
		*AUC_60_	460	0.758	0.001
	L5	AUC_240_	460	0.634	0.011
WA		AUC_60_	470	0.562	0.037
		*AUC_240_	470	0.745	0.002
		AUC_240_	480	0.599	0.039
		Min	490	0.654	0.011
		AUC_60_	490	0.640	0.019
		AUC_240_	420	0.572	0.041
	VL5	AUC_60_	450	0.573	0.032
		Min	460	0.556	0.039
	Fourier Pot_1/1-12_	Min	450	-0.577	0.050
		AUC_60_	470	-0.596	0.032
		*AUC_240_	420	0.691	0.009
Light		AUC_60_	460	0.563	0.036
	L5	AUC_240_	460	0.513	0.060
		Min	460	0.606	0.022
		AUC_240_	470	0.570	0.042

M5, the mid-point time of the five consecutive hours with the highest values; L5, the mid-point time of the five consecutive hours with the lowest values; VL5, value for L5; Min, minimum pupil diameter (expressed as pupil constriction); AUC_60_, AUC_240_, Area Under the Curve (inverted, expressed as pupil constriction) during the first minute (60 seconds) and the last minute (240 seconds) of light exposure.

* indicates statistical significance after Bonferroni correction.

For wrist activity, however, the later times of L5 and higher levels of activity during the sleep period (VL5) corresponded to an enhanced transient and sustained pupil response under wavelengths mainly from 460 to 490 nm. Robustness in the light exposure pattern as observed by Fourier Pot_1/1-12_ and both transient and intermediate pupil responses were negatively correlated, and again a later L5 time was associated with enhanced pupil constriction at both transient, intermediate and sustained responses, mainly under 460–470 nm light.

As shown in Table 5, the Horne-Östberg Morningness-Eveningness Questionnaire (H-O) score was negatively correlated with some of the selected PLR parameters, most of them obtained with light above 460 nm, indicating that the more morningness the participant exhibited, the more reduced pupil constriction both in the transient and sustained response. The Pittsburgh Sleep Quality Index (PSQI) score was positively correlated with the PLR parameters for most wavelengths longer than 450 nm, indicating that the worse the sleep quality, the more marked the transient and sustained pupil response. The Epworth Sleepiness Scale only showed a negative correlation with TL120 at 480 nm (*R* = -0.811, *p* = 0.004), but did not show any significant correlation with the previously selected PLR parameters (Min, AUC_60_, AUC_240_).

**Table 5:** Correlation between morningness-eveningness, and sleep quality questionnaires *vs*. PLR.

	PLR correlation			
Questionnaire	PLR parameter	Wavelength (nm)	R	*p*
H-O	*AUC_240_	420	-0.771	0.005
	AUC_240_	440	-0.638	0.035
	AUC_60_	460	-0.666	0.018
	AUC_240_	460	-0.619	0.032
	*AUC_300_	460	-0.747	0.005
	*AUC_240_	470	-0.757	0.007
	AUC_300_	470	-0.657	0.028
	AUC_60_	490	-0.592	0.055
	Min	480	0.671	0.017
	AUC_240_	450	0.697	0.017
PSQI	AUC_300_	450	0.619	0.042
	AUC_60_	460	0.626	0.029

H-O, Horne-Östberg Morningness-Eveningness score; PSQI, Pittsburgh Sleep Quality Index; Min, minimum diameter (expressed as pupil constriction); AUC_60_, AUC_240_, AUC_300_, Area Under the Curve during the first minute (0–60 seconds), last minute (240–300 seconds) of light exposure and the minute after light offset (300–360 seconds), respectively.

* indicates statistical significance after Bonferroni correction.

Correlations between the desynchronization among the different rhythmic variables (detailed in Materials and Methods) and the PLR parameters were also calculated, finding positive correlations, shown in Table 6. This finding indicated that higher desynchronization among the different rhythms was linked to a more marked transient and sustained response of the PLR.

**Table 6:** Correlation between Internal Desynchronization (DI) and PLR.

	PLR correlation			
DI	PLR parameter	Wavelength (nm)	R	*P*
WT/L	Min	460	0.514	0.050
	AUC_240_	440	0.581	0.038
	Min	440	0.569	0.027
WT/A	Min	460	0.559	0.030
	Min	490	0.533	0.050
	Min	500	0.640	0.014

WT/L, desynchronization between wrist temperature (WT) rhythm and light exposure pattern (L); WT/A, desynchronization between wrist temperature (WT) and motor activity (WA) rhythms. Min, minimum pupil diameter (expressed as pupil constriction); AUC_60_, AUC_240_, Area Under the Curve during the first minute (60 seconds) and the last minute (240 seconds) of light exposure. Only correlations with *p* ≤ 0.05 are shown although they did not reach statistical significance when Bonferroni correction was applied.

We proposed the CSI (Circadian Status Index) (Materials and Methods) as an integrative measure to unify three aspects (robustness, timing and level) of the three rhythmic variables studied together with other aspects such as diurnal preference and sleep quality (Horne-Östberg Morningness-Eveningness Questionnaire and Pittsburgh Sleep Quality Index, respectively). A global parameter for the pupil response was also calculated (Materials and Methods), including maximum constriction (or minimum diameter achieved after light onset), the sustained constriction (measured at the last minute of light exposure, AUC_240_) for two extreme wavelengths (420 and 500 nm) tested and for the optimal “circadian light”, at 460 nm. When this new integrative parameter for 420 nm (“PLR_420_”), 460 nm (“PLR_460_”) and 500 nm (“PLR_500_”) was compared with the CSI, only PLR_460_ showed a significant negative correlation (R = -0.519; *p* = 0.048), while PLR_420_ and PLR_500_ did not (R = -0.403; *p* = 0.136 and R = -0.369; *p* = 0.176, respectively), indicating that the greater pupil light responses (more marked transient and sustained responses) under 460 nm light were associated with a worse circadian status (less robustness).

**Fig 5:**
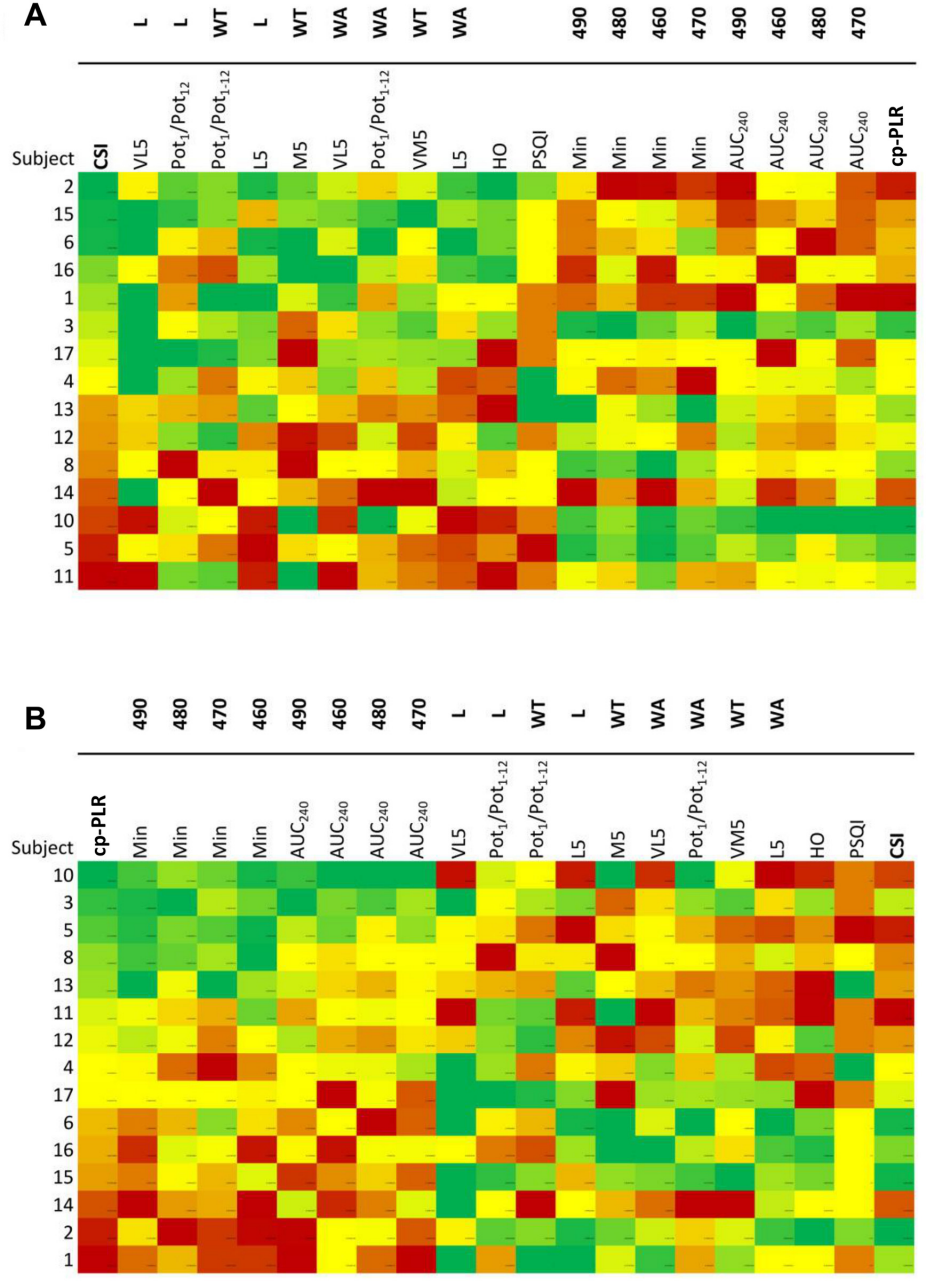
Macroarrays according to high and low cp-PLR and CSI parameters. Graphic matrix for subjects (n = 15) sorted by (A) CSI (circadian status index) and (B) by cp-PLR (circadian photoreception PLR). Colour scale corresponds to the relative magnitude for each variable and participant: reddish colours indicate lower scores for both CSI (worse circadian system status) and PLR parameters (less constriction) with those wavelengths in the circadian range (460-490nm). For details on the cp-PLR and CSI indexes calculation see Materials and Methods. The variables included in the macroarrays are wrist temperature (WT), wrist acceleration (WA) and light exposure (L) from the circadian analysis and normalized minimum pupil diameter expressed as pupil constriction and AUC_240_. M5, the mid-point time of the five consecutive hours with the highest values; VM5, value for M5; L5, the mid-point time of the five consecutive hours with the lowest values; VL5, value for L5; HO, Horne-Östberg Morningness-Eveningness Questionnaire score; PSQI, Pittsburgh Sleep Quality Index; Min, minimum pupil diameter (expressed as pupil constriction); AUC_240_, Area Under the Curve during the last minute (240 seconds) of light exposure. See Materials and Methods for the calculation details.

In order to obtain an integrative pupillary response parameter focussed on the “circadian” part of the spectrum (460–490 nm), the cp-PLR index (circadian photoreception PLR) (see Materials and Methods for details) together with the CSI, was used to classify participants according to their response. The cp-PLR index showed a significant negative correlation (R = -0.539; *p* = 0.038) with CSI, thus, the higher the pupil response under these wavelengths (460–490 nm), the worse the circadian status. Fig 5 shows the graphic matrix (macroarrays) for our study population ordered either by CSI (Fig 5A) or cp-PLR (Fig 5B). These figures show a colour gradient ranging from the worst pattern (low transformed and normalized values, in red) to the best pattern (green) for each circadian parameter variable, while for the PLR parameters, the red colour indicates less pupil constriction, and the green colour indicates greater pupil constriction. Thus, actual later times for M5 for WT, L5 for WA and L, and high values for VL5 for WA and L would be coloured red. Most participants showed a clear inverse correlation between their CSI and cp-PLR parameters, that confirms graphically the relationship between greater pupil responses under 460–490 nm light and worse circadian status.

## Discussion

Our results showed a consistent relationship between a greater pupillary response to monochromatic blue light and most of the rhythmic parameters associated with circadian disruption, such as low robustness, fragmentation, and eveningness, as calculated from wrist temperature, motor activity and light exposure patterns. These correlations were stronger in response to 460 nm light than to 420 or 500 nm light exposures. To the best of our knowledge, this is the first time that a close relationship between circadian system functionality and the pupillary response to light (PLR) has been described. Two new indexes to evaluate, in an integrative way, both circadian system status (CSI), and the PLR response (cp-PLR) have also been proposed.

Since PLR recordings yield a large amount of data, one of our aims was to propose an integrated parameter that could be used as a tool to assess global PLR under melanopsin-stimulating light wavelengths in relation to circadian system functioning under normal living conditions. The pupil response immediately after light onset mainly reflects the activity of the rods and cones (transient pupillary response), whereas the steady-state pupil size and post-illumination pupil response (PIPR) are more dependent on ipRGC activity, which is stimulated directly by light acting on melanopsin (intrinsic photoresponse) and indirectly by rods and cones (extrinsic pathway) [42–44]. Thus, to compose an integrated PLR parameter we selected, as representative parameters for the PLR, the minimum pupil diameter (indicative of the transient pupillary response) and the Area Under the Curve between 240–300 seconds, AUC_240_ (indicative of the sustained pupillary response). In a recent study, however, AUC has been found to exhibit high intra-individual variability with acceptable inter-individual variability [21]. In the present study, intra-individual variation was not assessed. In future, similar studies could be performed with our defined parameters to assess inter- and intra-individual variability. AUC_300_, although not included in the integrated cp-PLR parameter, was considered for some of the correlations. However, since the AUC_300_ was obtained from the minute of darkness immediately following the 5-minute light exposure, it may be that the pupil had already recovered its initial size in some individuals.

In a similar way, from all the parameters that can characterize the circadian system, we have selected one each for the following circadian characteristics: level, timing and robustness, as previously reported [25,26]. As a level measure, the mean values of the five consecutive hours of lowest activity, that is, the M5 value for skin temperature and the L5 value for motor activity and light exposure were selected, due to its close relationship with the sleep pattern [25,45]. Concerning circadian system timing, M5 (for wrist temperature) and L5 (for motor activity and light exposure) midpoints were chosen since they have shown a temporal association with melatonin onset (DLMO) [26]. For circadian robustness, the ratio between the power of the first circadian harmonic (24 h period) and the accumulated power of the first twelve circadian harmonics (until 2 h period) was used since this index is well correlated with the predominance of 24 h rhythm over ultradian components [26,41]. In addition to these objective measures, diurnal preference [30], sleep quality (PSQI) and daytime sleepiness (Epworth sleepiness scale) were used as subjective measures. These three traits (level, timing and robustness) for the three different circadian patterns (wrist temperature, activity, and light exposure) together with sleep quality and diurnal preference have been implemented into a single score named the Circadian Status Index (CSI) to facilitate global assessment of the circadian system.

Regarding correlations found between PLR and rhythmic variable levels, higher motor activity values during the rest period were positively correlated with more marked pupil constriction under the different light stimuli, suggesting that better rest consolidation (thus, less motor activity during the night), which could be associated with higher peripheral vasodilation, in a similar way as lower sleep onset latency [46], would be related to a reduced pupillary constriction response. This association could be a consequence of the influence of the autonomic nervous system balance towards a high parasympathetic tone that would favour deep sleep [47], thus higher sympathetic activity and lighter sleep would be related with more marked pupil constriction. Indeed previous studies have shown that pupillometry is more sensitive than other classical cardiovascular indexes for detecting neurovegetative tone, so much so it has been correlated with sleep apnoea (a condition associated with sympathetic activation during sleep) and autonomic dysfunction [48,49] and thus, pupillometry is considered a useful tool for assessing autonomic nervous system dysregulation.

Interestingly, positive correlations were found between circadian phase markers from the variables studied, namely M5 (WT) and L5 (WA and L) and the pupil response, that point to the later the timing of the rhythms, the greater the pupil constriction (smaller pupil size). In agreement, scores from the Morningness-Eveningness Questionnaire [30] also showed a significant negative correlation with the pupillary constriction parameters, thus evening types presented more marked pupil constriction. Interestingly, most of the light conditions that showed these correlations were around 460 nm (near the melanopsin λ_max_ peak of sensitivity). However, PLR parameters obtained using longer wavelength λ_max_480 nm light were not significantly correlated with questionnaires scores, probably due to data variability. The low coefficient of determination obtained could also indicate other non-controlled factors in this study since PLR can be significantly influenced by processes such as changes in accommodation states, in the state of arousal or even cognitive activity [16]. Thus although participants were instructed to refrain from caffeinated drinks, alcohol, excessive exercise, bright lights and non-steroidal anti-inflammatory drug intake, their strict compliance is impossible to ensure.

The PLR response could also be influenced by the time when the test was performed, since circadian and diurnal influence on the PLR has been reported [50]. These authors demonstrated that the intrinsic melanopsin system becomes less sensitive to light in the second half of the night, after the peak of melatonin production and closer to wake time, with the greatest pupil constriction occurring after a blue light stimulus (λ_max_ 463 nm) at the beginning of the day, and decreasing progressively afterwards [50]. Furthermore, as shown for the visual photoreceptive system, a temporal change in the sensitivity of the circadian photoreceptors has also been suggested [51], including a differential phase relationship between the predominantly melanopsin-mediated and the primarily cone-driven post-illumination pupil responses relative to the onset of melatonin secretion [52]. Thus, it could be hypothesized that circadian modulation via external, circulating, and/or central stimuli may have a differential effect on rod, cone, and ipRGC sensitivity, which may be detectable through the PLR.

In order to minimize the possible time of day and circadian effect on the PLR response, tests were performed exclusively during the morning, standardizing the timing for the first light condition in each session and for each participant. The high variability observed in the circadian phase markers among the participants, however, suggests that they had different circadian phases despite the consistency as to when the PLR test was performed. Thus although all the PLR tests were performed in the morning, differences in pupil constriction depending on a person’s internal phase cannot be discarded. The PLR of an evening type participant could therefore be more sensitive to light than a morning type participant, whose melanopsin response was far from its point of circadian maximal sensitivity.

Paradoxically, less pupil constriction (i.e. reduced PLR) was correlated, in general, with markers of rhythm robustness. According to our initial hypothesis, a robust circadian system should present a greater ipRGC response and consequently, higher sustained pupil constriction under 460–480 nm light exposure since the ipRGCs innervate both the olivary pretectal nucleus (OPN) involved in the PLR and the SCN clock. However, it should be considered that different subtypes of ipRGCs with different functional roles exist. Thus, although it has been described that both the SCN and OPN are innervated by the M1 ipRGC subtype, two distinct subpopulations of the M1 subtype have been reported, namely Brn3b^+^ and Brn3b^-^ [53]. Chen and colleagues (2011) concluded that both the M1 subpopulations, Brn3b^+^ and Brn3b^-^, morphologically and electrophysiologically similar, differentially innervate the OPN and the SCN, respectively. Consequently, the more Brn3b^+^ cells a person has, the more sustained pupil response would be present, but less Brn3b^-^ cells, with less projections to the SCN and less entrainment power of the light-dark cycle may occur. Thus, the apparent contradiction between circadian status robustness and the PLR response could be attributable to individual differences in the M1 cells population. This could be a possible explanation of our results, although it could also be hypothesized that as more light information reaches the OPN (with higher sustained pupil constriction), less photic information is available for SCN-driven entrainment, not only because of the different distribution of the ipRGC subpopulations, but also for light information use by the different neural pathways.

Insufficient light intensity cannot explain our results, since according to recent studies in primates, the photon flux threshold for ipRGC activation is ∼11 log quanta/cm^2^/s at 480 nm [16]. This photon flux was also sufficient to elicit the PLR in a blind person studied by Gooley et al. [44]. A recent study established the threshold at ∼10 log quanta/cm^2^/s for melatonin suppression under 460 nm monochromatic light [17]. Although this finding cannot be directly translated to the PLR, it could be relevant for our purpose in assessing the relationship between PLR and circadian status. In the present study, the photon fluxes tested ranged from 11.57 log quanta/cm^2^/s (500 nm) to 11.66 log quanta/cm^2^/s (440 nm) (correcting for optical media). Thus, all the light stimuli were applied above 11 log quanta/cm^2^/s, which is within the limits for melanopsin activation, although in the lower range described before [15]. Thus, regarding the light administration, two limitations of our study are *i)* the differences, although minimal, in photon flux between the different light conditions and the low photon fluxes achieved, due to technical limitations, and *ii)* the uncontrolled potential influence of bistability [54]. The order of light administration was randomized to minimize any bias.

The tested range of wavelengths in this study was selected based on previous studies in relation to the effect of light wavelength on melatonin production [34–38]. Although these data cannot be directly translated to the PLR, since our aim was to make a first approach to the possible relationship between the PLR and global circadian system status, to explore the possible usefulness of pupillometry to indirectly evaluate circadian status, the chosen wavelengths were specifically relevant. Moreover assessment of the relationship between circadian system status and PLR under light wavelengths shorter than those for melanopsin's peak sensitivity could help to assess the usefulness of replacing the blue (~ 480 nm) part of the spectrum in situations where illuminating during the night while maintaining chromaticity is necessary. However, more studies using appropriate protocols to isolate each photoreceptor contributions are needed to clarify the link between each photoreceptor activation and the circadian system status.

For the first time, the relationship between the PLR under different short wavelength blue monochromatic lights and circadian status assessment has been studied. The positive correlations found between a greater pupillary response to monochromatic blue light around 460 nm and circadian disruption, assessed from wrist skin temperature, motor activity and light exposure patterns, points to pupillometry being a suitable technique, not only to evaluate the integrity of the non-visual light pathways, but also to predict the circadian system status of people under free living conditions. Further studies are required to determine the mechanisms which may explain the consistent and robust associations between the pupil response and the circadian system status here reported.
